# Low-Frequency Repetitive Transcranial Magnetic Stimulation for the Treatment of Chronic Tinnitus: A Systematic Review and Meta-Analysis of Randomized Controlled Trials

**DOI:** 10.1155/2020/3141278

**Published:** 2020-05-02

**Authors:** Changhong Dong, Cheng Chen, Teng Wang, Chunjiu Gao, Yidan Wang, Xinying Guan, Xin Dong

**Affiliations:** ^1^Department of Neurology, The Affiliated Lianyungang Hospital of Xuzhou Medical University/The First People's Hospital of Lianyungang, 6 Zhenhua East Road, Lianyungang, Jiangsu Province, China; ^2^Department of Clinical Medicine, Xuzhou Medical University, Xuzhou 221004, Jiangsu Province, China; ^3^Department of Neurology, The First Affiliated Hospital of Nanjing Medical University, 300 Guangzhou Road, Nanjing, Jiangsu Province, China; ^4^College of Medical Imaging, Xuzhou Medical University, Xuzhou 221004, Jiangsu Province, China

## Abstract

**Background:**

Chronic tinnitus affects approximately 10-15% of the population. Low-frequency repetitive transcranial magnetic stimulation (rTMS) has been considered as a promising and well-tolerated therapeutic strategy for chronic tinnitus. However, a recent large-scale multicenter clinical trial showed a negative result.

**Objective:**

This systematic review is aimed at assessing the efficacy and safety of low-frequency rTMS in chronic tinnitus.

**Methods:**

We searched PubMed, Embase, and Cochrane Library for randomized controlled studies of rTMS treatment of chronic tinnitus. A pooled analysis of standardized mean difference (SMD) was performed with 95% confidence intervals (CI).

**Results:**

Ten RCTs involving 567 participants were included in this review. Compared with sham stimulation, rTMS showed no significant efficacy in tinnitus severity and disability measured by Tinnitus Handicap Inventory (THI) in short-term (SMD = −0.04, 95% CI -0.23 to 0.16, *P* = 0.72), medium-term (SMD = −0.13, 95% CI -0.43 to 0.17, *P* = 0.41), and long-term (SMD = −0.16, 95% CI -0.38 to 0.05, *P* = 0.14) follow-up. Tinnitus severity and disability measured by Tinnitus Questionnaire (TQ) also showed no significant improvement in short-term (SMD = −0.11, 95% CI -0.31 to 0.10, *P* = 0.30), medium-term (SMD = −0.10, 95% CI -0.37 to 0.16, *P* = 0.44), and long-term (SMD = −0.20, 95% CI -0.40 to 0.01, *P* = 0.06) follow-up. Additionally, no statistically significant difference was shown in the changes of tinnitus loudness assessed by a visual analogue scale (VAS) between rTMS and sham groups in the short-term (SMD = −0.28, 95% CI -0.59 to 0.02, *P* = 0.07), medium-term (SMD = −0.26, 95% CI -0.59 to 0.07, *P* = 0.13), and long-term (SMD = −0.20, 95% CI -0.53 to 0.13, *P* = 0.24) follow-up. Few mild or moderate adverse events were observed in both the rTMS and sham groups.

**Conclusion:**

Low-frequency rTMS is well tolerated but not effective in treating chronic tinnitus based on the current analysis of pooled data. Further studies with modified and uniform protocols are required to investigate the potential benefit of rTMS in chronic tinnitus.

## 1. Introduction

Tinnitus is a frequent auditory sensation characterized by the perception of noise without any external acoustic stimulus [1]. Chronic tinnitus affects approximately 10-15% of the adult population [2, 3] and severely impairs the quality of patients' daily life including attention, sleep, and emotional disorder. Several management strategies have been proposed for the treatment of tinnitus including pharmacological intervention, hyperbaric oxygen therapy, cognitive behavioral therapy, tinnitus masking, and music therapy [4–6]. However, these therapies are not satisfactory in all tinnitus patients and the evidence related to the efficacy of these therapies is inconclusive. There is an urgent need to find more effective therapeutic strategies for chronic tinnitus.

Transcranial magnetic stimulation (TMS) is a noninvasive neuromodulation technique that produces electrical currents in the brain based on the principle of electromagnetic induction [7]. Repetitive transcranial magnetic stimulation (rTMS) applies a train of repetitive pulses to alter the excitability of the neurons and modulate cortical activity [8]. High-frequency rTMS increases cortical excitability, while low-frequency rTMS is considered to inhibit the neural activity in stimulated regions [9]. Hence, low-frequency rTMS has been proposed as an innovative treatment strategy for tinnitus which is associated with hyperexcitability in the auditory cortex.

Several studies that applied rTMS for the treatment of chronic tinnitus patients have been carried out. Previous systematic reviews [10, 11] assessing the efficacy of rTMS treatment in chronic tinnitus have demonstrated that rTMS may provoke a beneficial effect on chronic tinnitus. However, the different study designs and stimulated patterns affect the stability and reliability of outcomes. A recent multicenter randomized controlled trial (RCT) with a large sample size has been published and found that low-frequency (1 Hz) rTMS over the temporal cortex is not superior to sham rTMS in reducing tinnitus severity [12]. Therefore, we performed a systematic review and meta-analysis of RCTs to investigate the effect of low-frequency rTMS in chronic tinnitus patients.

## 2. Materials and Methods

### 2.1. Study Design and Registration

This systematic review and meta-analysis was conducted on the basis of the Preferred Reporting Items for Systematic Reviews and Meta-analyses (PRISMA) statement. The protocol for this systematic review was registered at the International Prospective Register of Systematic Reviews (number CRD42018106368).

### 2.2. Search Strategy

We searched the electronic databases of PubMed, Embase, and Cochrane Library to find relevant studies published up to August 2019 with no language restrictions using the following search terms: “transcranial magnetic stimulation”, “Magnetic Stimulation, Transcranial”, “tinnitus”. We also searched the reference list of relevant articles. The complete search strategy can be found in Supplement 1.

### 2.3. Study Selection and Data Extraction

We included studies according to the following criteria: (1) participants diagnosed as subjective tinnitus, (2) performing low-frequency rTMS with figure-eight coil, (3) comparing real rTMS with sham rTMS, (4) quantitatively reporting the efficacy of rTMS on tinnitus severity and quality of life, and (5) randomized controlled trials. Exclusion criteria were (1) performing rTMS in combination with other interventions and (2) certain publications such as reviews, meta-analysis, letters, or case reports.

Two authors (Chen and Wang) independently scanned the retrieved abstracts and selected those possibly relevant articles for full-text reading. The two authors independently assessed the eligibility of the studies according to the inclusion and exclusion criteria. We resolved disagreements by discussion or by a third review author (Wang).

The following information was extracted independently by two authors: study characteristics (first author and publication year), subject characteristics (number of patients, age, gender, and baseline characteristics), intervention parameters (stimulation location and intensity and duration of treatment), outcome measurements, follow-up, and adverse effects. The primary outcomes were the improvement in tinnitus severity and disability assessed by validated tinnitus-specific questionnaires including Tinnitus Handicap Inventory (THI) and Tinnitus Questionnaire (TQ). The secondary outcomes were the change in tinnitus loudness measured with a visual analogue scale (VAS) and the adverse effects. When the data were incomplete or ambiguous from the publication, we turned to the corresponding authors for detailed data. Where outcomes were assessed at multiple time points, independent meta-analyses were performed in the short-term (within one week after treatment), the medium-term (one week to one month after treatment), and the long-term (longer than one month after treatment) follow-up.

### 2.4. Assessment of Risk of Bias

Two reviewer authors (Dong and Gao) independently assessed the risk of bias using the Cochrane Collaboration's Risk of Bias tool [13]. Disagreements were resolved by discussion or by a third reviewer (Chen). The Cochrane Collaboration's tool includes seven domains of bias: random sequence generation (selection bias), allocation concealment (selection bias), blinding of participants and personnel (performance bias), blinding of outcome assessment (detection bias), incomplete outcome data (attrition bias), selective reporting (reporting bias), and other bias.

### 2.5. Statistical Analysis

We used Review Manage software 5.3 (Cochrane Collaboration) to perform data analysis. The scores assessed by tinnitus-specific questionnaires were considered as continuous variables. For continuous outcomes, we calculated the standardized mean difference (SMD) with 95% confidence intervals (CI). Statistical heterogeneity between studies was tested using the Chi^2^ test and *I*^2^ statistic. *P* < 0.1 in the Chi^2^ test and *I*^2^ values greater than 50% were identified as significant heterogeneity. If no significant heterogeneity was found, we used a fixed-effects model to calculate pooled estimates of the treatment effect. Otherwise, a random-effects model was used. Sensitivity analysis was performed by excluding low-quality studies.

## 3. Results

### 3.1. Study Selection

**Figure 1:**
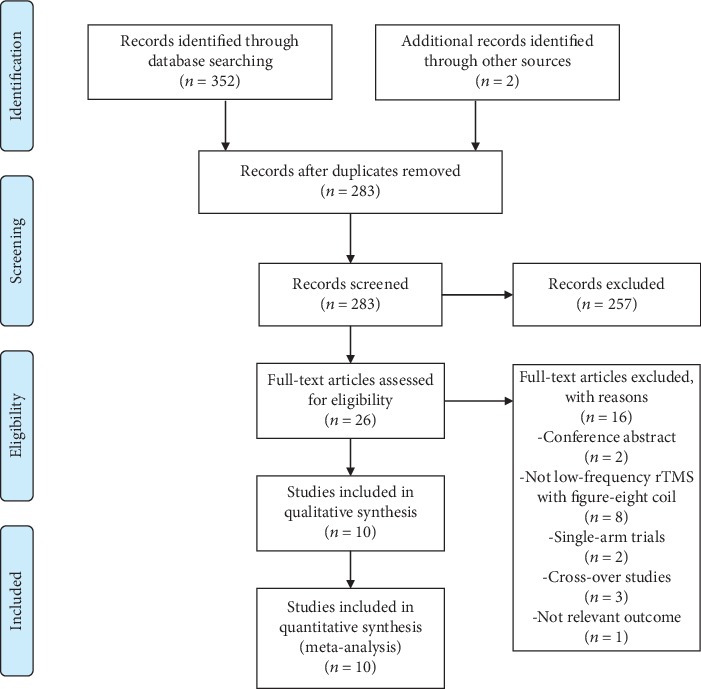
Flowchart for study selection process. rTMS = repetitive transcranial magnetic stimulation.

We retrieved 354 records through searches in the electronic database. Twenty-six articles remained when we removed the duplicates and screened the titles and abstracts. After screening the full text, we included 10 RCTs [12, 14–22] with a total of 567 participants in our meta-analysis (Figure 1).

### 3.2. Study Characteristics

The characteristics of the included studies are summarized in Table 1. These ten studies were all published between 2010 and 2019. All patients suffered from chronic tinnitus, and the duration of tinnitus was at least 1 month. All studies applied low-frequency rTMS to the temporal cortex, the temporoparietal cortex, or the temporal combined with the frontal regions. The duration of treatment ranged from 5 days to 10 days. The follow-up periods in these studies ranged from 1 week to 6 months after treatments. All trails compared the efficacy of rTMS with sham stimulation.

### 3.3. Risk of Bias

**Figure 2:**
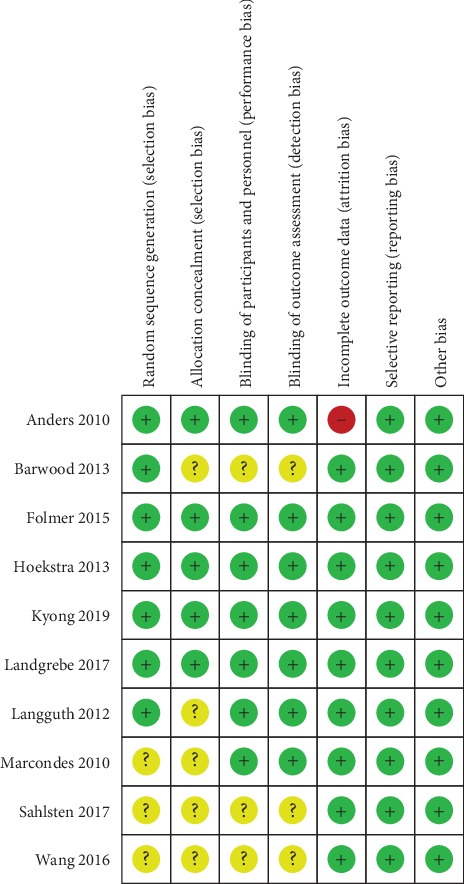
Risk of bias summary of included studies.

The risk of bias of the nine included studies is summarized in Figure 2. Seven studies [12, 14, 16–19, 21] described the method of random sequence generation in detail, while three studies [15, 20, 22] did not provide sufficient information about randomized methods. Five studies [12, 14, 16, 17, 21] reported adequate allocation concealment. Seven included studies [12, 14, 16, 17, 19–21] were explicitly designed as double blinded, and three studies [15, 18, 22] did not clearly describe the process of double blinding. It is difficult to achieve real blinding of personnel during the sham stimulation. Thus, double blinding referred to the blinding of participants and outcome assessment. Therefore, we deemed seven studies at low risk and three studies at unclear risk of performance and detection bias. All studies in this review provided reasons for the withdrawals. We considered only one study [21] to be at high risk of attrition bias due to the high dropout rate. We judged reporting bias and other bias as low in all studies.

### 3.4. Primary Outcomes

#### 3.4.1. Improvement in Tinnitus Severity and Disability

**Figure 3:**
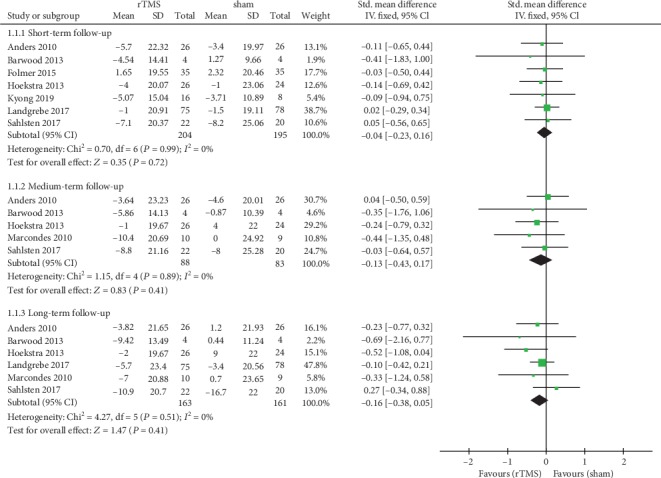
Meta-analysis of the effects of real rTMS on the changes of tinnitus severity and disability as measured by Tinnitus Handicap Inventory (THI).

All studies assessed the changes of tinnitus severity and disability after treatment using validated questionnaires, such as TQ or THI. Among these studies, eight studies [12, 14–18, 20, 21] with 418 participants assessed the efficacy of rTMS on tinnitus using THI. Pooled analysis showed that real rTMS treatment had no significant effect on THI compared with sham groups in the short-term follow-up (SMD = −0.04, 95% CI -0.23 to 0.16, *P* = 0.72), with no heterogeneity (*I*^2^ = 0%) (Figure 3). Five studies [15, 17, 18, 20, 21] provided data on changes of THI scores in the medium-term follow-up. Pooled analysis of the data showed no significant improvement in THI scores in the rTMS group compared with sham stimulation (SMD = −0.13, 95% CI -0.43 to 0.17, *P* = 0.41). There was no significant heterogeneity between studies (*I*^2^ = 0%) (Figure 3). Six studies [12, 15, 17, 18, 20, 21] assessed the effect of rTMS treatment on tinnitus severity and disability using THI in the long-term follow-up. There was no significant effect of rTMS on THI compared with sham groups in the long-term follow-up (SMD = −0.16, 95% CI -0.38 to 0.05, *P* = 0.14), with no heterogeneity (*I*^2^ = 0%) (Figure 3).

**Figure 4:**
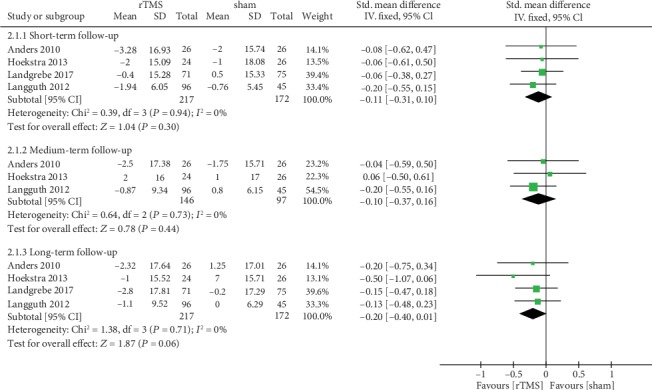
Meta-analysis of the effects of real rTMS on the changes of tinnitus severity and disability as measured by Tinnitus Questionnaire (TQ).

Four studies [12, 17, 19, 21] assessed the efficacy of rTMS on the tinnitus severity as measured by TQ. For the short-term effect after intervention, the pooled estimate of data showed that rTMS treatment led to no significant improvement in TQ scores compared with the sham group (SMD = −0.11, 95% CI -0.31 to 0.10, *P* = 0.30), with no heterogeneity (*I*^2^ = 0%) (Figure 4). In the medium-term follow-up, only three studies provided data on changes in scores measured by TQ. Pooling the data of these studies showed no significant difference between the rTMS group and the sham group (SMD = −0.10, 95% CI -0.37 to 0.16, *P* = 0.44) (Figure 4). Four studies included long-term follow-up data of TQ scores after rTMS treatment. The pooled results showed that there was no significant effect on the tinnitus severity as measured by TQ in the real rTMS group compared with the placebo (SMD = −0.20, 95% CI -0.40 to 0.01, *P* = 0.06) (Figure 4).

### 3.5. Secondary Outcomes

#### 3.5.1. Change in Tinnitus Loudness

**Figure 5:**
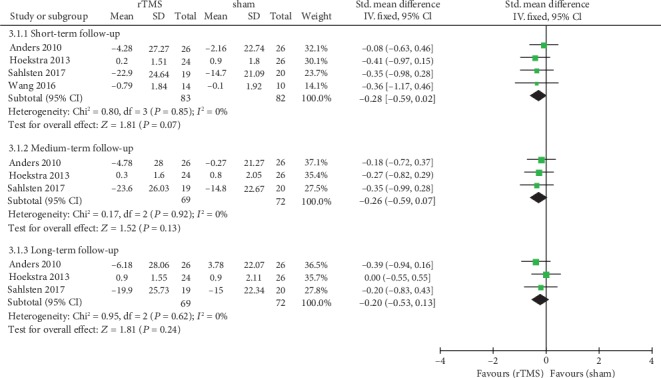
Meta-analysis of the effects of real rTMS on the change of tinnitus loudness measured with a visual analogue scale (VAS).

Four trials with 165 participants reported the change of tinnitus loudness after treatment using VAS. Pooled analysis demonstrated no statistically significant difference in the changes of tinnitus loudness assessed by VAS between rTMS and sham groups shortly after the intervention (SMD = −0.28, 95% CI -0.59 to 0.02, *P* = 0.07, *I*^2^ = 0%) (Figure 5). Only three studies reported data on the change of tinnitus loudness in the medium-term and long-term follow-up. For the medium-term effect, pooling data of the three studies did not show any benefit of low-frequency rTMS for tinnitus loudness compared with sham stimulation (SMD = −0.26, 95% CI -0.59 to 0.07, *P* = 0.13, *I*^2^ = 0%) (Figure 5). The pooled estimate of the long-term effect size of rTMS on VAS showed no significant benefit effect of rTMS (SMD = −0.20, 95% CI -0.53 to 0.13, *P* = 0.24) with a nonsignificant heterogeneity (*I*^2^ = 0%) (Figure 5).

#### 3.5.2. Adverse Effects

Adverse effects associated with rTMS treatment were reported in detail in four studies. Anders et al. [21] reported that one patient experienced a headache, two patients complained about the deterioration of their tinnitus, and one woman suffered from pain in the stimulation area and unpleasant muscle contraction in the neck in the rTMS group. Two cases of headache and three cases of worsening of tinnitus were reported in the sham group. Langguth et al. [19] reported headache, site discomfort, and facial twitching in about 15% of the patients treated with real rTMS and in 7% of the sham-treated patients. In the study by Hoekstra et al. [17], five patients reported headaches in the rTMS group and one patient experienced a headache in the sham group. Landgrebe et al. [12] reported one severe adverse effect in both groups. One patient with a known cardiac insufficiency experienced tachyarrhythmia in the rTMS group, and one case of severe headache and deterioration of tinnitus was recorded in the sham group.

## 4. Discussion

This systematic review including 10 RCTs shows that low-frequency rTMS did not provide any benefit for chronic tinnitus patients when compared with the placebo. Low-frequency rTMS is safe and well tolerated.

The findings of our review are inconsistent with the previous meta-analysis [10, 11]. Our meta-analysis included four recently published RCTs, especially one [12] multicenter study with a large sample size. Contrary to our results, the previous systematic review by Soleimani et al. stated the moderate efficacy of rTMS for chronic tinnitus. Soleimani et al. [11] included studies with various stimulation procedures such as low-frequency, high-frequency, and theta burst, while our meta-analysis only included studies investigating the effects of low-frequency rTMS on tinnitus. What is more, the effects of rTMS treatment at different time points, such as follow-up periods in the short term, medium term, and long term, were separately estimated in our meta-analysis.

One possible factor related to the negative results is the limited number of rTMS sessions. The treatment duration of two weeks for tinnitus might be too short to induce positive clinical benefit [23]. Clinical trials using rTMS to treat depression have supported positive clinical efficacy of rTMS after four weeks of treatment, rather than two weeks [24–26]. We may infer that tinnitus patients might also benefit from rTMS with longer treatment duration. Another possible explanation is that the stimulation target may not be optimal. In most of the included studies, the stimulation coil was positioned over the left auditory cortex. Nevertheless, other studies found that the treatment of rTMS over the temporoparietal cortex contralateral to the side of tinnitus has a more significant beneficial effect on tinnitus than the left side stimulation.

In addition, only four studies used the neuronavigation system to guide TMS coil placement to achieve an optimal location of the patients' primary auditory cortex. In other studies, the rTMS coil was positioned over the temporal cortex or temporoparietal cortex using a 10–20 EEG-system. The estimates of rTMS effects on tinnitus could be confounded by differences in coil positioning to the location of the patients' primary auditory cortex between studies [22, 27]. What is more, we performed the pooled analysis of tinnitus severity detected by THI and TQ during treatment and follow-up. One systematic review that assessed six questionnaires of evaluating treatment outcomes in tinnitus patients stated that those questionnaires may not be sufficient and proper to measure the effectiveness of rTMS intervention [28]. Meikle et al. demonstrated that the questionnaire of Tinnitus Functional Index (TFI) is more useful to measure the treatment responsiveness in tinnitus for clinical and research settings [29]. Finally, variant placebo procedures are used in these included studies, such as applying a sham coil without producing a magnetic field or tilting the coil 45° or 90° away from the midline. However, the coil tilted 45° or 90° may create a weak magnetic field [30] that may affect the excitability of cortical neurons [16]. Thus, a possible therapeutic response to sham stimulation may lead to the absence of a significant superiority in treatment outcomes for real stimulation [31]. There is an urgent need for an adequate placebo condition for studies which may be of limited value on account of patients' awareness of differences between sham and real stimulation [32].

Several limitations exist in our meta-analysis. A limitation of this meta-analysis is that the parameters for rTMS stimulation including the location, the side, and the duration are varied in included RCTs, and thus, the optimal stimulation protocol remains uncertain. There is an urgent need for further research to propose a consistent protocol. Secondly, our meta-analysis only evaluates the efficacy of low-frequency rTMS treatment in tinnitus. However, this cannot exclude the possible benefit of other rTMS stimulation procedures since high-frequency stimulation and theta burst stimulation have been demonstrated to be effective in chronic tinnitus in other studies. Thirdly, the sample size of most included studies ranging from 8 to 64 patients was relatively small. Of the included trials, there are only two RCTs with large sample sizes of 146 and 151 patients. Additionally, nearly half of the studies did not provide the methods of randomization and allocation concealment, reducing the quality of evidence of outcomes in this review due to the unclear risk of bias in included studies. Finally, we did not perform subgroup analysis based on the stimulation target due to the limited number of included trials.

## 5. Conclusion

Low-frequency rTMS should not be recommended for routine clinical use based on the current evidence of this review. Our results should be interpreted with caution due to the inherent limitation of the included studies. Further large-volume and well-designed studies are needed to investigate the potential effect of rTMS treatment in chronic tinnitus.

## Figures and Tables

**Table 1:** Characteristics of included studies on repetitive transcranial magnetic stimulation for tinnitus in the meta-analysis.

Study	Groups(*n*)	Gender (male/female)	Age (years)	Patients	Stimulation location	Parameters	Treatment duration	Follow-up	Measurement
Anders 2010, [21]	Real: 26 Sham: 26	Real: 12/10 Sham: 17/3	Real: 48.09 ± 14.86 Sham: 50.05 ± 13.97	Tinnitus > 6 months	Left primary auditory cortex	1 Hz, 110% RMT, 1500 pulses	2 weeks	Posttreatment, 1 month, 6 months	THI, TQ, VAS
Marcondes 2010, [20]	Real: 10 Sham: 9	Not reported	>18 years old	Tinnitus > 3 months	Left temporoparietal cortex	1 Hz, 110% RMT, 1020 pulses	5 days	1 month, 6 months	THI
Langguth 2012, [19]	Real: 96 Sham: 45	Real: 67/29 Sham: 31/14	Real: 50.4 ± 12.5 Sham: 50.3 ± 12.9	Tinnitus > 3 months	Left temporal cortex	1 Hz, 110% RMT, 2000 pulses	2 weeks	Posttreatment, 7 weeks, 11 weeks	TQ
Barwood 2013, [18]	Real: 4 Sham: 4	Real: 2/2 Sham: 2/2	Real: 40.25 ± 4.92 Sham: 44.5 ± 13.03	Tinnitus > 1 month	Left primary auditory cortex	1 Hz, 100% RMT, 2000 pulses	2 weeks	1 week, 1 month, 3 months	THI
Hoekstra 2013, [17]	Real: 26 Sham: 24	Real: 26/0 Sham: 15/9	Real: 50 ± 12 Sham: 55 ± 12	Tinnitus > 2 months	Bilateral primary auditory cortex	1 Hz, 110% RMT, 2000 pulses	5 days	Posttreatment, 1 month, 6 months	THI, TQ, VAS
Folmer 2015, [16]	Real: 32 Sham: 32	Real: 25/7 Sham: 26/6	Real: 58.3 ± 9.5 Sham: 62.8 ± 8.3	Tinnitus > 1 year	Left or right auditory cortex	1 Hz, 110% RMT, 2000 pulses	2 weeks	Posttreatment	THI
Wang 2016, [22]	Real: 14 Sham: 10	Real: 6/8 Sham: 3/10	Real: 62.1 ± 9.81 Sham: 56.4 ± 11.8	Tinnitus > 6 months	Left temporoparietal cortex	1 Hz, 110%MT, 1000 pulses	2 weeks	Posttreatment	VAS
Landgrebe 2017, [12]	Real: 71 Sham: 75	Real: 54/17 Sham: 51/24	Real: 48.1 ± 12.5 Sham: 49.9 ± 13.2	Tinnitus > 6 months	Left temporal cortex	1 Hz,110% RMT, 2000 pulses	2 weeks	Posttreatment, 6 months	THI, TQ
Sahlsten 2017, [15]	Real: 19 Sham: 20	27/12	Real: 48.9 ± 13.1 Sham: 51.5 ± 10.7	Tinnitus > 6 months	Left auditory cortex	1 Hz,100% RMT, 4000 pulses	2 weeks	1 week, 1 month, 6 months	THI, VAS
Kyong 2019, [14]	Real: 16 Sham: 8	Real: 10/6 Sham: 6/2	Real: 56.4 ± 7.4 Sham: 50.9 ± 7.1	Tinnitus > 6 months	Temporal cortex or the temporal and the frontal regions	1 Hz,110% RMT, 3000 pulses	2 weeks	Posttreatment	THI

Real = real group; sham = sham group; RMT = resting motor threshold; THI = Tinnitus Handicap Inventory; TQ = Tinnitus Questionnaire; VAS = visual analogue scale.

## Supplementary Materials

Supplementary Materials Supplement 1: literature search strategies.
