# Supplementary material for: Low-Frequency Repetitive Transcranial Magnetic Stimulation for the Treatment of Chronic Tinnitus: A Systematic Review and Meta-Analysis of Randomized Controlled Trials
Source: Biomed Res Int. 2020 May 2;2020:3141278. doi: 10.1155/2020/3141278 (PMC7218966; doi:10.1155/2020/3141278)
Supplement: Supplementary Materials — Supplement 1: literature search strategies. [file 3141278.f1.docx]

**Search strategy for Pubmed**
#1 "Tinnitus"[Mesh] 
#2 tinnit* [Title/Abstract] 
#3 (ear* [Title/Abstract] AND (buzz* [Title/Abstract] OR ring* [Title/Abstract]OR roar* [Title/Abstract] OR click* [Title/Abstract] OR puls* [Title/Abstract])) 
#4 #1 OR #2 OR #3 
#5 "Transcranial Magnetic Stimulation"[Mesh] 
#6 (Transcranial Magnetic Stimulation[Title/Abstract]) OR Magnetic Stimulation, Transcranial[Title/Abstract]

#7 #5 OR #6

#8 "randomized controlled trial"[pt] OR "controlled clinical trial"[pt] OR randomized[tiab] OR placebo[tiab]  OR randomly[tiab] OR trial[tiab] OR groups[tiab]
#9 #4 AND #7 AND #8

**Search strategy for Embase**
#1 'tinnitus'/exp

#2 'tinnit*':ab,ti

#3 (ear AND (buzz* OR ring* OR roar* OR click* OR puls*)).ti.

#4 #1 OR #2 OR #3

#5 'transcranial magnetic stimulation'/exp

#6 'transcranial magnetic stimulation':ab,ti OR 'tms':ab,ti OR 'rtms':ab,ti

#7 #5 OR #6

#8 'randomized controlled trial':ab,ti OR 'random':ab,ti OR 'placebo':ab,ti OR

'trial':ab,ti

#9 #4 AND #7 AND #8

**Search strategy for Cochrane Library**
#1 MeSH descriptor: [Tinnitus] explode all trees
#2 tinnit*:ti,ab,kw (Word variations have been searched)
#3 #1 OR #2
#4 MeSH descriptor: [Transcranial magnetic stimulation] explode all trees
#5 Transcranial magnetic stimulation:ti,ab,kw (Word variations have been searched)
#6 #4 OR #5 
#7 #3 AND #6
